# Polyvinyl Alcohol/Nafion^®^–Zirconia Phosphate Nanocomposite Membranes for Polymer Electrolyte Membrane Fuel Cell Applications: Synthesis and Characterisation

**DOI:** 10.3390/membranes13120887

**Published:** 2023-11-23

**Authors:** Rudzani Sigwadi, Fulufhelo Nemavhola

**Affiliations:** 1 Department of Chemical Engineering, University of South Africa, Roodepoort 1710, South Africa; 2 Department of Mechanical Engineering, Faculty of Engineering and the Built Environment, Durban University of Technology, Durban 4000, South Africa; fulufhelon1@dut.ac.za

**Keywords:** PVA (polyvinyl alcohol), zirconia phosphate, glutaraldehyde (GA), water crosslinking agent, mechanical strength

## Abstract

PVA (polyvinyl alcohol)-ZrP (PVA/ZrP) and Nafion^®^/PVA-ZrP nanocomposite membranes were synthesised using the recasting method with glutaraldehyde (GA) as a crosslinking agent. The resulting nanocomposite membranes were characterised using a variety of techniques, including X-ray diffraction (XRD), scanning electron microscopy (SEM), and Fourier transform infrared spectroscopy (FTIR). The results of SEM revealed well-distributed zirconia phosphate (ZrP) within the membrane matrix, and the SEM images showed a uniform and dense membrane structure. Because ZrP nanoparticles are hydrophilic, the Nafion^®^/PVA-ZrP nanocomposite membrane had a higher water uptake of 53% at 80 °C and higher 0.19 S/cm proton conductivity at room temperature than the commercial Nafion^®^ 117 membrane, which had only 34% and 0.113 S/cm, respectively. In comparison to commercial Nafion^®^ 117 membranes, PVA-ZrP and Nafion^®^/PVA-ZrP nanocomposite membranes had a higher thermal stability and mechanical strength and lower methanol crossover due to the hydrophilic effect of PVA crosslinked with GA, which can make strong hydrogen bonds and cause an intense intramolecular interaction.

## 1. Introduction

When compared to other fuel cell types, polymer electrolyte membrane fuel cells (PEMFCs) have the highest power densities [1]. They have an exceptional chemical, mechanical, and thermal stability, as well as a high proton conductivity when they are hydrated. The most-used type of polymeric membrane for PEMFCs is perfluorosulfonic acid (PFSA) membranes, such as Nafion^®^ [2]. However, Nafion^®^ membranes have a significant drawback of dehydrating at temperatures above 80 °C, resulting in a reduced proton conductivity and mechanical stability [3].

Other ion-conductive membranes, like Aquivion; inorganic–organic, crosslinked, water-based membranes; and Nafion^®^ membranes blended with other polymers have been introduced using a variety of techniques. Among these ion-conductive membrane polymers, Aquivion was found to demonstrate a higher performance than Nafion NRE-211 and can operate at a higher temperature of up to 120–140 °C [4]. However, several challenges were encountered during the commercial manufacturing of Aquivion membranes. Due to these challenges leading to higher costs in producing the polymer [4], the researchers chose to use existing Nafion^®^ (Ion Power, New Castle, DE, USA) products.

Polyvinyl alcohol (PVA) is a synthetic polymer that is biocompatible, inexpensive, and has excellent film-forming and adhesive properties as well as a high tensile strength and flexibility [5,6] and a high potential for chemical crosslinking. In contrast, PVA has a low proton conductivity and a high swelling rate [7], making it suitable for use in aqueous environments for a variety of applications, including water treatment [8]. One way to overcome the water solubility issue is by crosslinking the PVA molecules [9] to create a network structure that is insoluble in water. Crosslinked PVA can be used in various applications, such as in the production of hydrogels, coatings, and membranes [10]. Furthermore, its excellent film-forming and adhesive properties make it easy to blend with other polymers [2]. Moreover, when Nafion^®^ membranes and PVA membranes are used together, the amount of Nafion^®^ solution required decreases, resulting in lower fabrication costs, less methanol permeation, and an increased water resistance.

According to research, the performance of the membrane can be improved by including hygroscopic metal oxide nanoparticles like SiO_2_ and TiO_2_ in the PVA polymer matrix [11]. Additionally, PVA’s water uptake and proton conductivity are enhanced by crosslinking it with aldehydes and dialdehydes as well as by combining it with other polymers, like Nafion^®^ and SPEEK [12]. Zirconium phosphate (ZrP) nanoparticles can enhance the performance of the membrane by improving its thermal and mechanical stability, as well as increasing its proton conductivity [13]. The PVA component provides good mechanical stability, while the Nafion^®^ component offers a high proton conductivity.

In this study, we altered PVA’s characteristics by combining it with a Nafion^®^ membrane and incorporating zirconium phosphate (ZrP) nanoparticles, which enhance the performance of the membrane by improving its thermal and mechanical stability, as well as increasing its proton conductivity. Thus, the goal of the current work is to assess the impact of ZrP nanoparticle incorporation on the blended polyvinyl alcohol (PVA) with a Nafion^®^ membrane in relation to their morphologies resulting from the blending system used. Polyvinyl alcohol (PVA)/Nafion^®^/ZrP nanocomposite membranes are promising candidates for use in PEMFCs.

## 2. Experimental

### 2.1. Chemicals

The following chemicals were used exactly as supplied: methanol (Sigma, Aston Manor, South Africa), zirconium oxychloride hydrate (Sigma), sulfuric acid (Merck, Merck Life Science (Pty) Ltd., Modderfontein, South Africa), hydrogen peroxide (Sigma), polyvinyl alcohol (99% hydrolysed, average Mw = 145,000, Merck), glutaraldehyde (25 wt% solution in water, Merck), dimethyl sulfoxide (DMSO) ≥ 99% (Sigma), D521 alcohol-based 1100 EW at 5 wt% Nafion^®^ solution, (Ion Power, Sigma-Aldrich (PTY) LTD, Aston Manor, South Africa), Nafion^®^ 117 membrane (Sigma), sodium hydroxide (Sigma), sodium chloride (Sigma), phenolphthalein (Sigma), and phosphoric acid (Sigma).

### 2.2. Synthesis of Zirconia Phosphate (ZrP) and Nanocomposite Membrane

The zirconia phosphate (ZrP) nanoparticles were prepared by adding 120 mL of 0.4 M ZrOCl_2_·8H_2_O aqueous solution to 6 M solution of phosphoric acid (H_3_PO_4_) and stirring it for 30 min. The solution was then refluxed at 80 °C for a further 24 h while stirring. The obtained material was then centrifuged and washed extensively with distilled water to pH 3 and dried at 80 °C and then calcined at 600 °C for two hrs [3].

The recasting method was used to synthesise PVA-ZrP and Nafion^®^/PVA-ZrP nanocomposite membranes. The membranes were synthesised by dissolving 15 wt% solution of PVA in dimethyl sulfoxide (DMSO) and water (1:1 *v*/*v*) solution. The mixture was stirred and heated at 70 °C to obtain a clear solution. ZrP nanoparticles were slowly added to the solution. To crosslink the membrane, 7 mL of glutaraldehyde (GA) was slowly added to the PVA-ZrP solution while stirring at 70 °C for 24 h. The homogeneous, viscous solution was divided into two parts. One part was transferred to petri dishes and labelled as PVA-ZrP. The Nafion^®^ solution was slowly added to the remaining PVA-ZrP solution while stirring at 70 °C for 4 h and then it was transferred to petri dishes and labelled as Nafion/PVA-ZrP. PVA-ZrP and Nafion^®^/PVA-ZrP solutions were dried at 60 °C for 24 h and the membrane dried for 1 h at 120 °C to make the crosslinking reaction. The membranes were then easily removed from the glass petri dishes and kept in DI water until the experiments were conducted. A boiling 3% hydrogen peroxide solution, boiling 0.5 M sulphuric acid, and boiling distilled water were used to treat Nafion^®^ 117 membranes for an hour each [5]. After drying, the thicknesses of the nanocomposite membranes were measured with digital micrometres (~0.18 mm). Each thickness was measured in the average of 3–7 readings at different positions of the membrane, and the process was repeated twice on each membrane to obtain the average value.

#### 2.2.1. Characterisations

The samples were continuously scanned from 10° to 90° while Rigaku Smartlab X-ray diffraction was performed with Cu K α radiation, λ = 1.54 Å. Under a nitrogen flow, Perkin Elmer thermal gravimetric analysis (TGA) and derivative thermo-gravimetric (DTG) analysis were used to examine the samples’ characteristics and thermal properties. With a heating rate of 10 °C/min from 28 °C to 1000 °C, TGA data were obtained using a TGA instrument (PerkinElmer, Shelton, CT, USA) over nitrogen. Using the JSM-7800F (Musashino, Akishima, Tokyo, Japan) field emission electron microscope (FE-SEM) model with a cathodoluminescence spectrometer, the surface morphologies of every membrane were examined. To investigate the changes in the chemical composition of the membranes, a Bruker Fourier transform infrared spectroscopy (FTIR) instrument was used.

#### 2.2.2. Tensile Test

The mechanical strength of the membranes was measured using a uniaxial testing system. A Vernier caliper was used to measure the breadth, thickness, and length. The clamping areas of all membranes (4 of each kind) were 4 mm × 10 mm in dimension. The measured thickness of ~0.18 mm was used to calculate the sample’s tension. Using the CellScale Ustretch Instrument, (CellScale Biomaterials Testing, Weber St. N. Waterloo, ON, Canada) membranes were measured at 25 °C at 10, 20, 30, and 40 mm/min actuator speeds. The use of multiple strain rates in membranes testing allows for a more complete understanding of a membrane’s behaviour under different conditions, and variations in elasticity values can be attributed to the rate-dependent nature of materials or membranes.

#### 2.2.3. Water Uptake (WU)

At different temperatures of 80 °C, 60 °C, and 30 °C for a period of 24 h, the membranes were submerged in distilled water before being measured and weighed. The water uptake and swelling ratio of the membrane after being submerged in water were calculated using the equations below:

(1) $$W_{up}=\frac{\left(m_{wet}-m_{dry}\right)}{m_{dry}}\times 100\%$$

m_dry_ and m_wet_ are the membrane wet and dry mass, respectively, and W_up_ is the water uptake percentage.

(2) $$SR=\frac{\left(L_{w}-L_{d}\right)}{L_{d}}\times 100\%$$

m_dry_ is the membrane dry mass, L_w_ is the membrane wet length, and L_d_ is the dried length of the membrane.

#### 2.2.4. Ion Exchange Capacity (IEC)

Based on the titrated results, the equation below was used to calculate the IEC of membranes:

(3) $$\mathrm{IEC}=\frac{V_{\mathrm{NaOH}\;}{\times \;C}_{\mathrm{NaOH}}}{m_{d}}$$

The volume of titrated NaOH is V_NaOH_, and the membrane dried mass is m_d_.

#### 2.2.5. Measurements of the Water Contact Angle

A contact angle measurement device (Phoenix 300 contact angle analyser—Surface Electro Optics, Kromtek Sdn Bhd (557373D), Shah Alam, Malaysia) with a video system was used to determine the hydrophilicity of the membrane surfaces. To ensure stability during the analysis, the membranes were cut into strips and attached to glass slides. A 0.16 μL droplet of deionised water was carefully placed on the membrane’s surface. This was performed by bringing the syringe’s tip up against the sample surface to ensure the precise and controlled application of the water droplet. The measurements were conducted at ambient temperature. The measurement process was repeated 20 times at different locations on the membrane surface to obtain multiple data points and ensure that the results are representative of the entire surface. A significant change at the surface could no longer be seen after a certain period of observation. The purpose of recording the wetting process is to capture the dynamics of the droplet’s interaction with the membrane surface, allowing researchers to measure the contact angle accurately. After obtaining the contact angle measurements from the 20 repetitions, the average value was calculated. The average value is used to represent the overall hydrophilicity of the membrane surface [6].

#### 2.2.6. Measurements of the Methanol Permeability

A permeation-measuring cell with two compartments that was created in our lab was used to measure the methanol permeability. Both 50 mL of a methanol solution and 50 mL of distilled water were placed in compartments A and B, respectively. The membrane was positioned in between the two compartments and had a 3.5 cm diameter for the diffusion area. The readings were taken with 5 M and 2 M methanol solutions at 30 °C, 60 °C, and 80 °C. The permeability (P) of methanol was calculated using the following equation:

(4) $$C_{B}=\frac{A\;P}{V_{B}\;L}\;C_{A}\;(t-t_{o})$$

where *C_A_* stands for the methanol in compartment *A*, *V_B_* for the volume of distilled water, *A* for the effective permeating area, and *L* for the membrane thickness; *C_B_*(*t*) is the amount of methanol in compartment *B* at time *t*.

#### 2.2.7. Measurement of the Proton Conductivity

**Figure 1:**
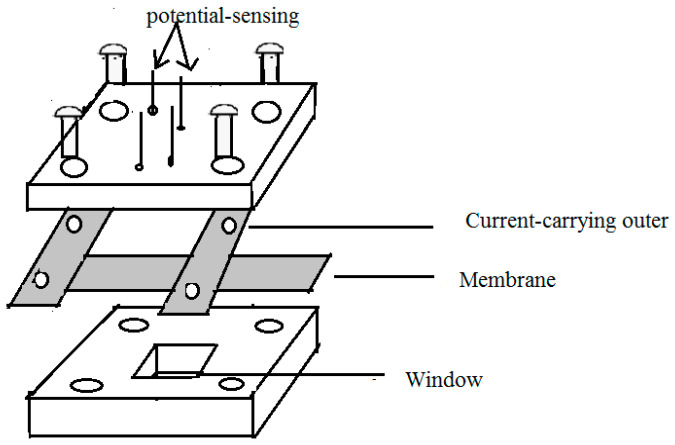
Schematic diagram of a proton conductivity cell for the four-point-probe electrochemical impedance spectroscopy technique.

Figure 1 illustrates the four-point collinear probe method used to measure the conductivities of commercial Nafion^®^ 117 membranes and nanocomposite membranes. An Autolab PGSTAT302 (Autolab, Basel, Switzeland) potentiostat/galvanostat in galvanostatic mode with an AC current amplitude of 0.1 mA was used to measure the ionic conductivity over a frequency range of 1 MHz to 10 Hz. The frequency range over which the impedance had a constant value was examined using a Bode plot, and the electrical resistance was then determined from a Nyquist plot. The equation below was used to determine the ionic conductivity (*k*):

(5) k=L/RWd

where *W* and *d* represent the membrane’s width (2 cm) and thickness, respectively, and *R* represents the measured membrane resistance. *L* represents the distance between the potential-sensing electrodes. The membrane was immersed in a 1 M sulfuric acid solution for 6 h at room temperature to test its conductivity. After the membrane was rinsed multiple times with deionised water to eliminate any remaining H_2_SO_4_, it was immersed in the deionised water for 6 h at 60 °C. Before the measurement, every membrane was stored at room temperature in deionised water.

## 3. Results and Discussion

### 3.1. Fourier Transform Infrared

**Figure 2:**
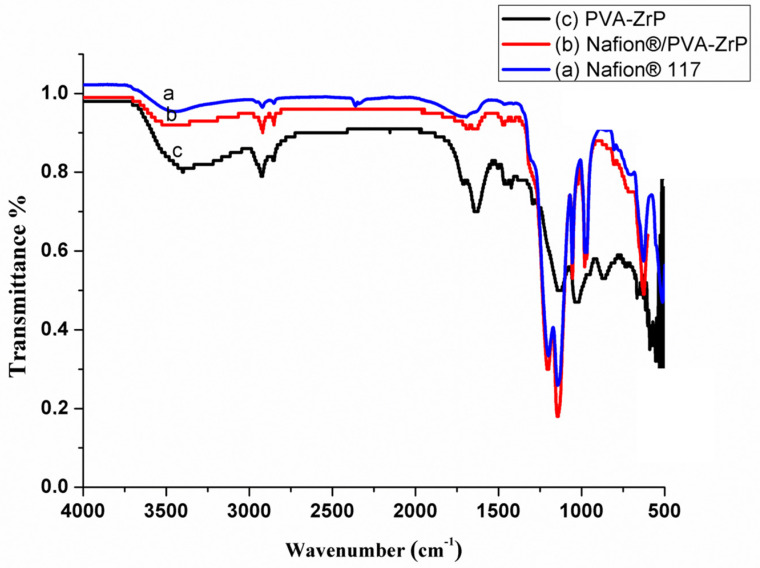
FTIR spectra of Nafion^®^ 117 membrane and Nafion^®^/PVA-ZrP and PVA-ZrP nanocomposite membranes.

FTIR spectra of Nafion^®^ 117 membranes and crosslinked PVA-ZrP and Nafion^®^/PVA-ZrP nanocomposite membranes are shown in Figure 2. The corresponding spectra of nanocomposite membranes crosslinked and modified with zirconia phosphate nanoparticles (Nafion^®^/PVA-ZrP) show that the spectral pattern of the membrane differs only slightly, and nearly all the distinctive peaks of the commercial Nafion^®^ membrane can be seen, as shown in Figure 2. The PVA-ZrP nanocomposite membranes in Figure 2 show a band around 3447 cm^−1^. The observed peak is attributed to the Nafion^®^ membrane’s—OH groups’ free O-H stretching vibration. The O-H stretching vibration from the intramolecular and intermolecular hydrogen bonds between the hydroxyl groups of the PVA membrane is attributed to the band at 3380 cm^−1^ in Figure 2 [7,8]. Figure 2 shows a peak at 2920 cm^−1^ due to the C-H stretching vibrations from alkyl groups. As shown in Figure 2, the band of PVA-ZrP nanocomposite membranes at 1710 cm^−1^ is attributed to the C=O and -C-O stretching from the remaining acetate groups in the PVA matrix [8,9]. The adsorbed water’s O-H bending is responsible for the absorbance peak at 1633 cm^−1^ seen in Figure 2. As seen in Figure 2, the bending vibration of the C-H bonds has been linked to the vibration peaks found at 1465 cm^−1^ and 1396 cm^−1^. The bands at 1396 cm^−1^ arise due to CH–OH [10]. The peak at 1135 cm^−1^ is assigned to the CO stretching mode, as shown in Figure 2. The intensity of the peak in the region of 1028 cm^−1^ increased with the addition of ZrP nanoparticles to the PVA matrix because of the contribution of C-O-C, as shown in Figure 2. This indicates that PVA and ZrP nanoparticles may interact. When ZrP nanoparticles are used, the composites also exhibit less of the C-H’s rocking vibration at 867 cm^−1^ [11], which is quite noticeable. It is discovered that the ZrP nanoparticles added to the PVA matrix have a negligible impact on the degree of O-H stretching. This might be because the ZrP nanoparticles’ surface hydroxyl groups interact with the nearby hydroxyl groups within the PVA matrix.

### 3.2. Membrane Morphology

**Figure 3:**
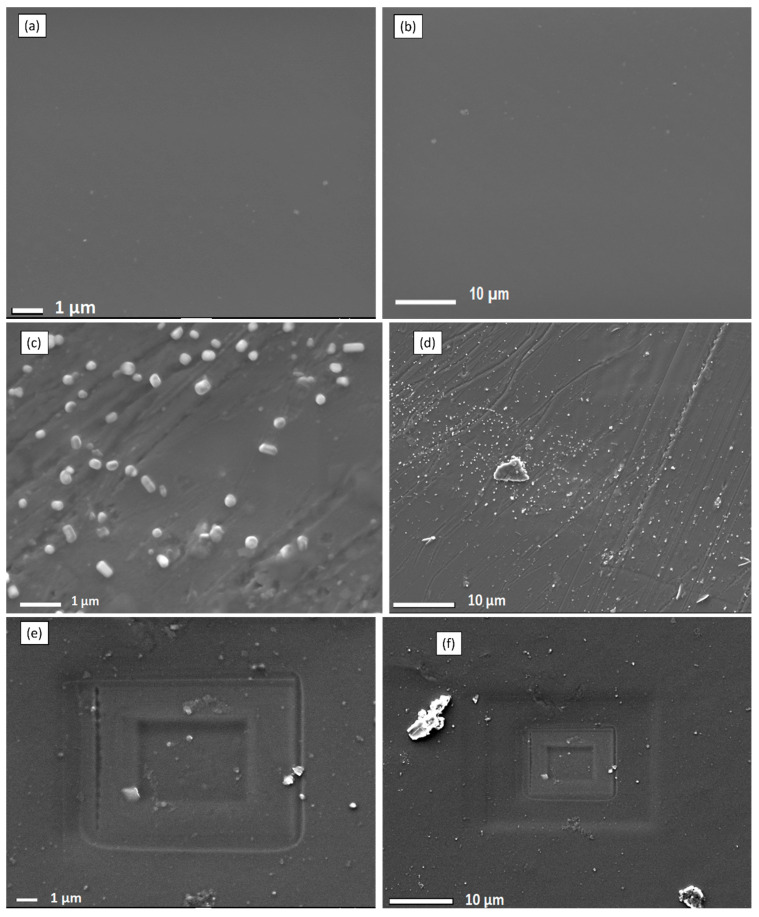

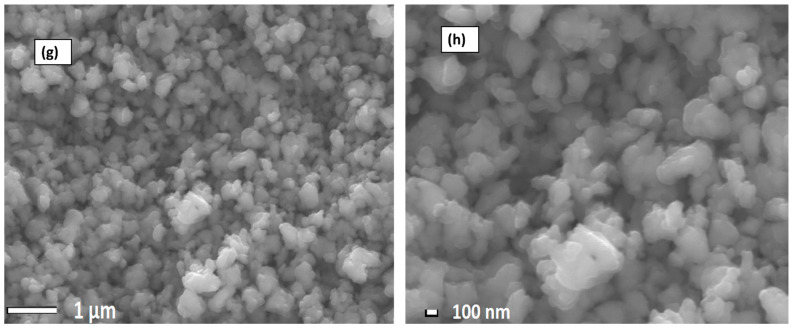
SEM images of Nafion^®^ 117 membranes (**a**,**b**), PVA-ZrP membranes (**c**,**d**), Nafion^®^/PVA-ZrP nanocomposite membranes (**e**,**f**), and ZrP nanoparticles (1 µm and 100 nm) (**g**,**h**).

Figure 3 displays the SEM results of the Nafion^®^ 117 membrane and crosslinked PVA-ZrP and Nafion^®^/PVA-ZrP nanocomposite membranes. The Nafion^®^ 117 membrane is dark and free of nanoparticles, as shown in Figure 3a,b. According to Figure 3c,d, ZrP nanoparticles are well distributed throughout the linear PVA homogeneous phase. As shown in Figure 3c,d, the agglomerated nanoflakes of various sizes seem to take the shape of small nanorods inside the membranes. This might be because zirconia phosphate in nanoflake shape was well incorporated into the membrane during the process of recasting the crosslinked PVA membrane. The crosslinked Nafion^®^/PVA-ZrP appears to be remote in Figure 3e,f, because the PVA’s -OH groups are already used to create crosslinks and are therefore unavailable to create hydrogen bonds. It can also be seen that the ZrP nanoparticles are well dispersed within the Nafion/PVA matrix because they appear diffused and there is no obvious interface boundary between the matrix and the dispersed phase [12]. SEM results of ZrP nanoparticles are shown in Figure 3g,h. As seen in Figure 3g,h, the SEM image of ZrP nanoparticles reveals a spherical and nanoflake shape with less agglomeration in the size range of 100–150 nm. Furthermore, zirconia phosphate nanoparticles that are nanometres in size can be used in nanocomposite membranes to enhance the proton conductivity and decrease methanol permeability, which makes them more appropriate for use in PEMFCs.

**Figure 4:**
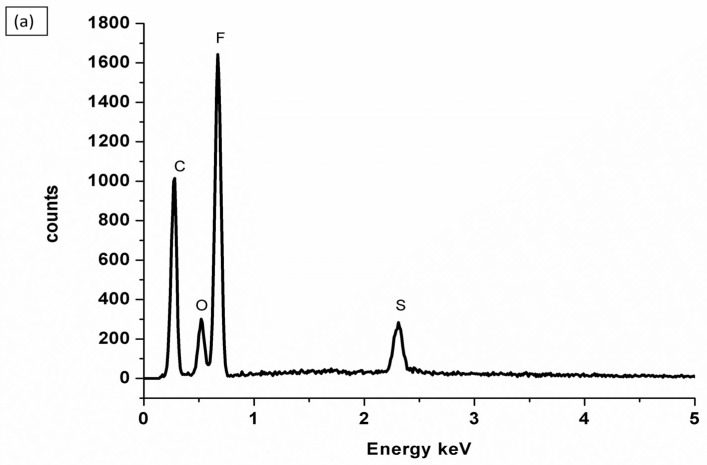

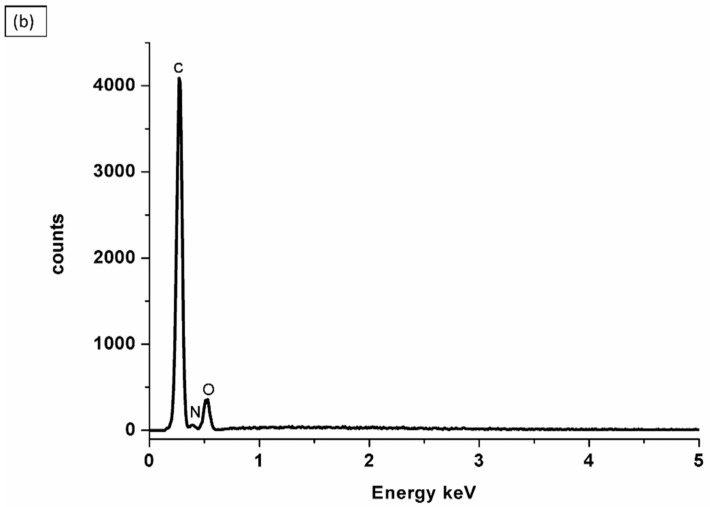
EDXs of nanocomposite membranes, Nafion^®^/PVA-ZrP (**a**) and PVA-ZrP (**b**).

EDX (Energy-Dispersive X-ray) analysis is a common analytical technique used to determine the elemental composition of materials. EDX elemental peaks guided by increased peaks of C, O, F, and S indicate that a Nafion^®^ membrane was successfully blended with PVA. This suggests that the EDX analysis detected higher levels of carbon (C), oxygen (O), fluorine (F), and sulfur (S) in the material. These elements are indicative of the presence of Nafion^®^ and PVA, as presented in Figure 4a. This is also evident in the SEM images of Nafion^®^/PVA-ZrP in Figure 3e,f, which also look porous. However, the impregnation with ZrP nanoparticles was not detected on the EDX; this may be due to the small size of the ZrP nanoparticles, which could not produce strong X-ray signals that can be detected by the EDX. The PVA-ZrP membrane contains oxygen, nitrogen, and carbon as the major elements, as shown in Figure 4b.

### 3.3. XRD Structure Analysis

**Figure 5:**
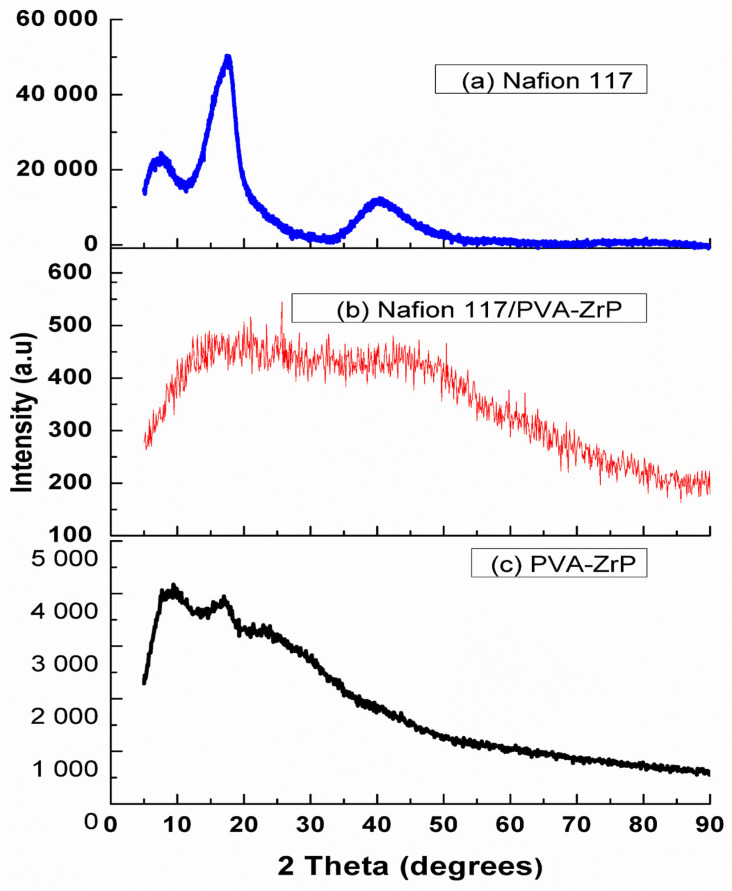
XRD images of Nafion^®^ 117 membrane (**a**), Nafion^®^/PVA-ZrP (**b**) and PVA-ZrP (**c**) nanocomposite membranes.

The XRD patterns for the Nafion^®^ 117 membrane and PVA-ZrP and Nafion^®^/PVA-ZrP nanocomposite membranes are shown in Figure 5 [12]. Figure 5a shows that the Nafion^®^ 117 membrane has two diffraction peaks at 17.5° and 39° 2θ, which can be assigned to the semi-crystallisation of the ionomer’s perfluorocarbon chains [13]). In the case of the blended PVA with Nafion^®^ membrane incorporated with 5 wt.% ZrP, it show a slight shift at a peak of 20.8°, which correspond to planes (2 0 0), as shown in Figure 5b [14]. The presence and weight percentage of ZrP nanoparticles may be responsible for the observed shift in the main chain of the PVA and Nafion^®^ membrane, resulting in a more amorphous structure. The findings from TGA and SEM further support the modifications to the membrane structure. Figure 5c shows the PVA-ZrP results, which exhibit the single scattering peak characteristic of PVA with a sharp peak at a 2θ value of 19.4° [12]. The weak peak at 2θ of 24.9 ° is attributed to the presence of crystalline peaks of ZrP, as shown in Figure 5b,c. The crosslinked nanocomposite membranes display a small peak of 2theta = 20.8. This may be due to the incorporation of ZrP nanoparticles.

### 3.4. Thermo-Gravimetric Analysis (TGA) and Derivative Thermo-Gravimetric (DTG) Analysis

**Figure 6:**
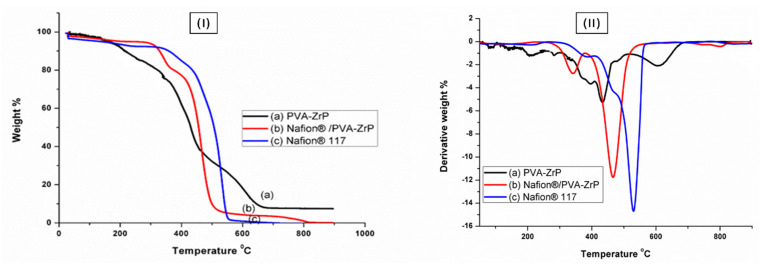
(**I**) The TGA and (**II**) DTG of PVA-ZrP and Nafion^®^/PVA-ZrP nanocomposite membranes and Nafion^®^ 117 membrane.

Figure 6I and II shows the TGA and DTG curves of the Nafion^®^ 117 membrane, and PVA/ZrP and Nafion^®^/PVA-ZrP nanocomposite membranes. The curves represent the weight loss of the samples as a function of temperature. The weight loss is caused by the thermal decomposition of the samples. Figure 6I shows that PVA-based nanocomposite films containing zirconia phosphate as a nanofiller have less weight loss than the Nafion^®^ 117 membrane, as shown in Figure 6I. The initial weight loss of all samples occurs in the temperature range of about 30–100 °C, as shown in Figure 6I. This weight loss is attributed to the evaporation of weakly bound moisture on the surfaces of the composite films. It was shown that PVA-ZrP started losing physically adsorbed water at 30 °C, as shown in Figure 6I. It was observed that linear PVA started losing adsorbed water almost at the same temperature in agreement with that previously reported [15]. The second weight loss occurred at around 200–400 °C, and this loss is due to the distribution of the inorganic filler [16]. Due to the development of hydrogen bonds between the PVA matrix and the added ZrP nanoparticles, the decomposition temperature in this range was elevated. A mixture of carbon and hydrocarbons, such as n-alkanes, n-alkenes, and aromatic hydrocarbons, was produced as a result of the PVA nanocomposite cleaving and the decomposition of carbonaceous materials, such as polyene residues, above 400 °C. Figure 6I shows the thermal stability of crosslinking Nafion^®^/PVA-ZrP, with weight loss in the range of about 5 wt.%, which is lower than the PVA/ZrP (20 wt.%) at 350 °C. Figure 6I shows the different weight loss stages observed in the thermal degradation of the Nafion^®^ 117 membrane. At 100 °C, the commercial Nafion^®^ 117 membrane in Figure 6I initially loses weight due to the evaporation of adsorption bound water to the sulfonic groups. Sulfonic group degradation could be responsible for the second weight loss at 380 °C. The third weight loss at 550 °C could be attributed to polymer backbone chain degradation [5,17]. In conclusion, reducing the mobility of the Nafion^®^ chain delays the initial loss of weight and thermal degradation of modified membranes when compared to the commercial membrane. This might be because the Nafion^®^ membrane contains water-retentive zirconia nanoparticles [18], which slow down weight loss and raise the temperature at which nanocomposites decompose.

Figure 6II displays the DTG analysis of crosslinked PVA-ZrP and Nafion^®^/PVA-ZrP membranes and the commercial Nafion^®^ 117 membrane, with a greater resistance to degradation, especially in the second stage, and greater amounts of particles at elevated temperatures have been noticeable. The DTG analysis in Figure 6II shows that nanocomposite membranes have a better heat stability than commercial Nafion^®^ 117, with nanocomposite membranes exhibiting a better thermal stability up to 340 °C, whereas Nafion^®^ 117 exhibits a better thermal stability up to 240 °C. The difference in thermal stability is attributed to the use of inorganic nanofillers in the nanocomposite membranes, which serve as a better mass transport barrier and insulator for volatile compounds created during decomposition. Peaks of the crosslinked Nafion^®^/PVA-ZrP nanocomposite’s DTG curve have shifted to the right, indicating the increased thermal stability of the PVA with the Nafion^®^ nanocomposite over the PVA-ZrP membrane. The thermal stability of modified PVA with Nafion^®^ membranes and inorganic nanofillers (ZrP) can make them suitable in PEMFC applications.

### 3.5. Tensile Tests

**Figure 7:**
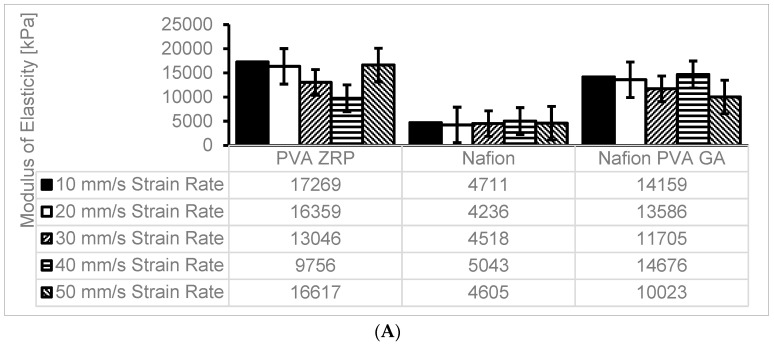

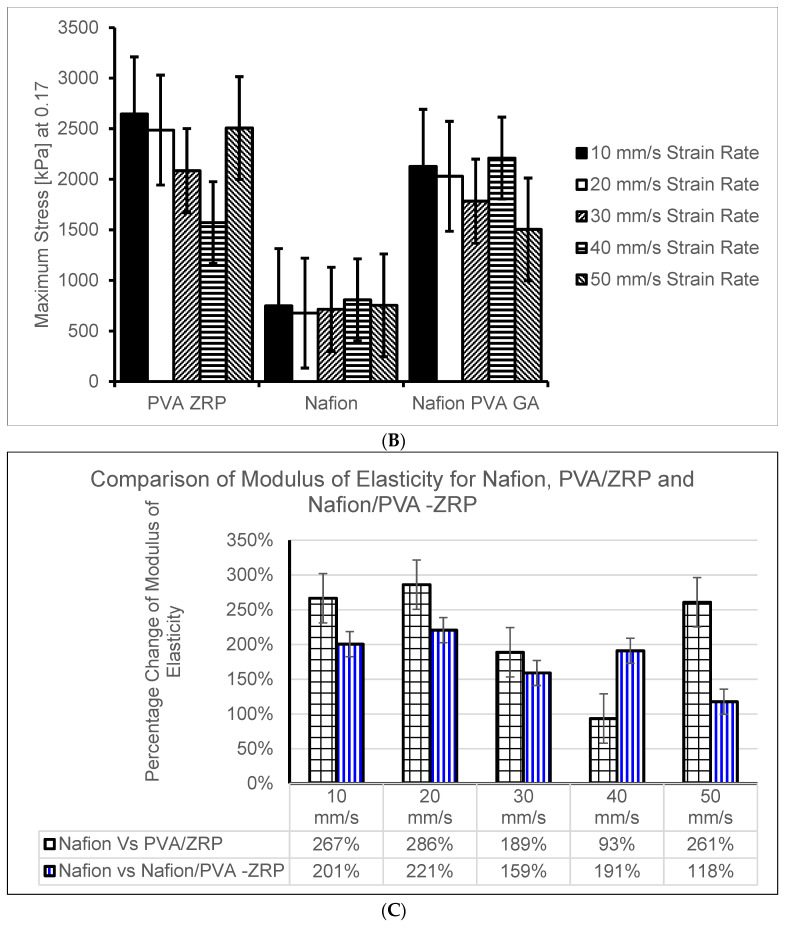
(**A**) Modulus of Elasticity at different strain rate for PVA-ZrP, Nafion^®^ and Nafion^®^/PVA-ZrP. (**B**) Maximum stress (kPa) computed at different strain rate for PVA-ZrP, Nafion^®^ and Nafion^®^/PVA-ZrP. (**C**) Comparison percentage comparison of Modulus of Elasticity for PVA-ZrP, Nafion^®^ and Nafion^®^/PVA-ZrP.

The Young’s modulus, or modulus of elasticity, is a measurement of a material’s stiffness and capacity to tolerate deformation in response to applied stress. The mechanical strengths of the commercial Nafion^®^ 117 membranes and the nanocomposite membranes were compared using the tensile test [19]. The modulus of elasticity was used to compare three materials, namely, the Nafion^®^ 117 membrane and PVA-ZrP and Nafion^®^/PVA-ZrP membranes (See Figure 7A–C). The results show that the incorporation of the ZrP nanofiller within the crosslinked PVA-ZrP and Nafion^®^/PVA-ZrP membranes enhances the modulus of elasticity based on the strain rates shown in Figure 7A–C at 10, 20, 30, 40, and 50 mm/s compared to the commercial Nafion^®^ 117 membrane. This may be due to the inorganic nanofiller (ZrP) dispersion within the PVA matrix and the strength of the interfacial adhesion between the two components improving the modulus of elasticity, since stronger interfacial adhesion and better dispersion lead to higher moduli. As shown in Figure 7C, crosslinked PVA-ZrP (17,000 kPa) and Nafion^®^/PVA-ZrP (14,000 kPa) membranes with a ZrP nanofiller have higher moduli of elasticity at strain rates of 10 mm/s than commercial Nafion^®^ 117 (4700 kPa). As can be seen in Figure 7B,C, the results showed that PVA-ZrP and Nafion^®^/PVA-ZrP nanocomposite membranes exhibit greater improvements in stress–strain. The degree of enhancement in tensile strength is greater in the crosslinked PVA/ZrP and Nafion^®^/PVA-ZrP nanocomposite membranes compared to the commercial Nafion^®^ membrane because the crosslinking creates stronger intermolecular bonds between the polymer chains [12] and enhances the rigidity or excellent flexibility [20]. Furthermore, the PVA-ZrP composite membrane’s modulus of elasticity is significantly impacted by crosslinking due to the covalent bonds formed between polymer chains (PVA) through the crosslinking process, which improve the mechanical properties of the material. The commercial Nafion^®^ 117 membrane has obtained a very low mechanical strength when compared to PVA-ZrP and Nafion^®^/PVA-ZrP membranes. Figure 7A–C shows that, irrespective of the strain rate, the mechanical properties of PVA-ZrP and Nafion^®^/PVA-ZrP membranes have swooned to outperform the Nafion^®^ 117 membrane. Figure 7C shows that the modulus of elasticity for PVA-ZrP has increased by 267% when compared to Nafion^®^ 117 at the 10 mm/s strain rate. This was the highest improvement of the modulus of elasticity when PVA-ZrP was compared with Nafion^®^ 117. This may be due to the type of membranes that behave more like a rigid material with a higher elastic modulus at higher strain rates, meaning they resist deformation more strongly [21]. PVA-ZrP and Nafion^®^/PVA-ZrP membranes exhibit greater deformability at lower strain rates, resembling more elastic behaviour. Additionally, at a 50 mm/s strain rate, the modulus of elasticity for the PVA-ZrP increased by 261% when compared to Nafion^®^ 117. However, when comparing Nafion^®^ 117 and Nafion/PVA-ZrP, it has been shown that the modulus of elasticity of Nafion/PVA -ZrP increased by 221% at 20 mm/s (See Figure 7C). This is because the presence of DMSO prevented free H_2_O molecules from solvating PVA chains, which strengthened the hydrogen bonding interactions that formed between DMSO and H_2_ [22].

### 3.6. Contact Angle Measurement

**Figure 8:**
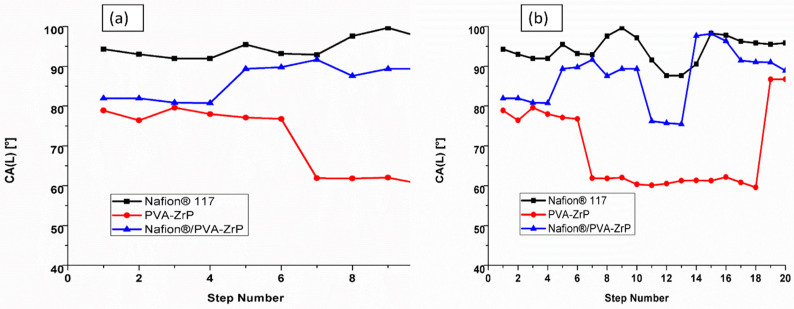
The water contact angle (**a**,**b**) of PVA-ZrP, Nafion^®^/PVA-ZrP nanocomposite membranes and Nafion^®^ 117 membrane.

The water contact angle of the commercial Nafion^®^ 117 membrane, PVA-ZrP and Nafion^®^/PVA-ZrP nanocomposite membranes were observed. The water droplets at the surface area of the nanocomposite membranes compared with the commercial Nafion^®^ 117 membrane is presented in Figure 8a,b. The increases water contact angle is related to the hydrophobic characteristic of Nafion^®^ 117 membrane, due to the presence of the sulphonic acid group in the hydrophilic side chain. As indicated in Figure 8a,b shows the higher water contact of 94° for Nafion^®^ 117 membrane compared with the reduced water contact of 85° and 69° for Nafion^®^/PVA-ZrP and PVA-ZrP nanocomposite membranes; this may be due to the hydrophilicity of zirconia phosphate nanoparticles and crosslinking. The decreased contact angles of nanocomposite membranes (below 90°), may due to the zirconia nanoparticles that are adsorbed on the surface of the membrane to enhance the hydrophilicity [23] and crosslinking process which reduces the ability of the PVA chains to dissolve in water [15]. Contact angles below 90 ° indicate the hydrophilic character of a material describing the water-uptake capability. A smaller contact angle less than 90° indicates good wettability and hydrophilicity, while a larger contact angle above 90° suggests poor wettability and hydrophobicity.

### 3.7. Methanol Permeability

As shown in Table 1, different methanol concentrations (2 M and 5 M) were used to compare the methanol permeability across the crosslinked PVA-ZrP and Nafion^®^/PVA-ZrP membranes to the commercial Nafion^®^ 117 membrane. Within a 2 h window, methanol samples were collected with the water bath set to 30 °C, 60 °C, and 80 °C. The obtained results indicated that there was no methanol permeation for any of the membranes at 2 M methanol. This may be because the methanol solution’s lower concentration may lower methanol permeation [24,25]. The methanol permeability increased when the methanol solution was increased to 5 M, as shown in Table 1. At a higher concentration of 5 M and a higher temperature of 60 °C, the commercial Nafion^®^ 117 membrane’s methanol permeability was 8.8 × 10^−7^ cm^2^/s higher than 0 cm^2^/s for PVA-ZrP and Nafion^®^/PVA-ZrP membranes, as shown in Table 1. Table 1 shows that, at 80 °C, the methanol permeability was measured to be 1.01 × 10^−6^ cm^2^/s, 1.25 × 10^−6^ cm^2^/s, and 1.98 × 10^−6^ cm^2^/s for PVA-ZrP and Nafion^®^/PVA-ZrP nanocomposite membranes and the Nafion^®^ 117 membrane, respectively. The PVA-ZrP and Nafion^®^/PVA-ZrP nanocomposite membranes obtained a low methanol permeability; this may be due to their chemical crosslinking and the incorporation of ZrP nanoparticles, which results in a beneficial interaction, leading to the membranes with extremely low methanol permeations. Furthermore, crosslinked PVA membranes incorporated with ZrP nanoparticles make them ideal for use in direct methanol fuel cells (DMFCs).

### 3.8. Water Uptake, Swelling Ratio, Ion Exchange Capacity, and Proton Conductivity Measurement

**Figure 9:**
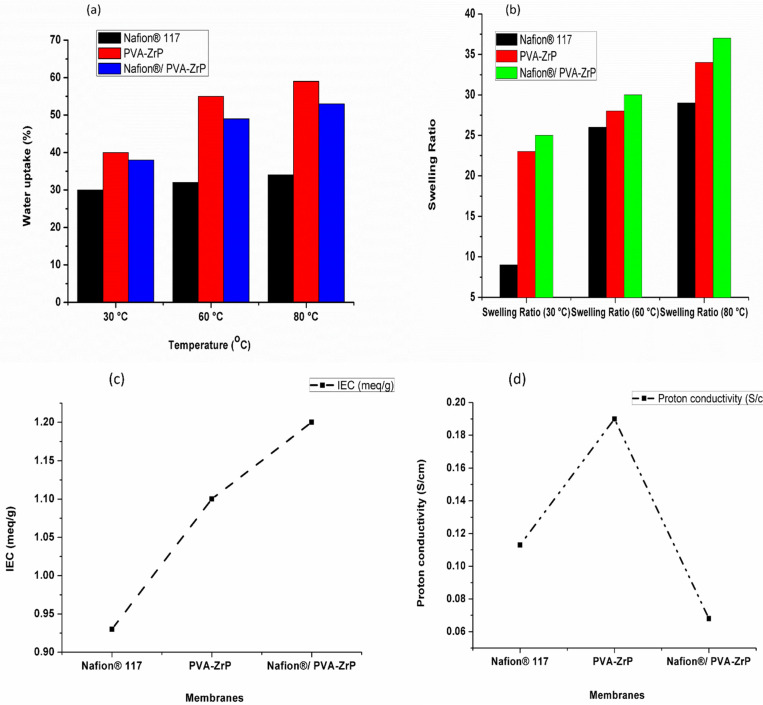
Measurement of Nafion^®^ 117 membrane and Nafion^®^/PVA-ZrP and PVA-ZrP nanocomposite membranes for water uptake (**a**), swelling ratio (**b**), ion exchange capacity (**c**), and proton conductivity (**d**).

The measurement of the water molecule content of a proton-exchange membrane is essential because the water content affects the membrane’s proton conductivity and overall performance in proton exchange membrane fuel cells (PEMFCs). Figure 9a and Table 2 show the water uptake of the Nafion^®^ 117 membrane and PVA-ZrP and Nafion^®^/PVA-ZrP nanocomposite membranes under various temperatures of 30 °C, 60 °C, and 80 °C. The water uptakes for crosslinked PVA-ZrP (59%) and Nafion^®^/PVA-ZrP (54%) nanocomposite membranes at 80 °C are higher than the commercial Nafion^®^ 117 membrane (34%) [17]; this implies that the crosslinked PVA membrane can absorb more water molecules than the Nafion^®^ 117 membrane. A higher water uptake enhances proton conductivity and improves the membrane’s overall performance in low-humidity environments [26].

As shown in Figure 9b, measurements of the swelling ratio of the Nafion^®^ 117 membrane and PVA-ZrP and Nafion^®^/PVA-ZrP membranes were made at 30 °C, 60 °C, and 80 °C. At 80 °C, the Nafion^®^ 117 membrane and PVA-ZrP and Nafion^®^/PVA-ZrP nanocomposite membranes exhibit swelling ratios of 29%, 34%, and 37%, respectively. As shown in Figure 9b, these membranes expand as the temperature rises; so, as the temperature rises, the membranes tend to swell more. At a higher temperature of 80 °C, the Nafion^®^ membrane blended with crosslinked PVA (Nafion^®^/PVA-ZrP) achieved a higher swelling ratio of 34% than the commercial Nafion^®^ 117 membrane (29%), as shown in Table 2. This is because the chemically crosslinked PVA has a hydrogel-like quality and may indicate that this mix of materials is more susceptible to swelling at high temperatures [27]. As the temperature rises, these membranes experience an increased dimensional stability as well as an increased dimensional swelling and water uptake. This may indicate that as the temperature rises, the membranes can take up more water. The results in Table 2 show that the nanocomposite membranes (PVA-ZrP and Nafion^®^/PVA-ZrP) have a better dimensional stability compared to the Nafion^®^ 117 membrane, particularly at higher temperatures of 80 °C. This improved stability might be due to the presence of ZrP nanoparticles within the composite structure.

Table 2 and Figure 9c show the IEC for the Nafion^®^ 117 membrane and PVA-ZrP and Nafion^®^/PVA-ZrP nanocomposite membranes. Equation (3) was used to calculate the IEC measurement. On the membranes, an acid-based titration was performed. The dried membranes were soaked in 2 M NaCl solutions to convert sulfonic acid to sodium. After the solution liberated the H^+^ ions from the membrane sample, 0.1 M NaOH was used to titrate it. After adding the phenolphthalein indicator drops, the NaOH solution’s volume and pH were measured [28]. The PVA-ZrP and Nafion^®^/PVA-ZrP nanocomposite membranes show ion exchange capacities of 1.1 and 1.2 meq/g, which are greater than the commercial Nafion^®^ 117 membrane’s 0.93 meq/g ion exchange capacity. As a result of ZrP nanoparticles being loaded onto the PVA and Nafion^®^ matrix, the blended membranes’ IEC values exhibit good agreement with water uptake values. Due to the presence of sulfonic acid groups in the polymer structure, the IEC values of the blended membranes increased [29]. Moreover, this may be due to the incorporation of metal oxide, which raises the sulfonate ions (SO_3_H) in the Nafion^®^ membrane and the acidic site within the Nafion^®^ and PVA matrix.

Table 2 and Figure 9d present the proton conductivity for the Nafion^®^ 117 membrane and PVA-ZrP and Nafion^®^/PVA-ZrP nanocomposite membranes. The proton conductivity of the crosslinked and blended Nafion^®^/PVA-ZrP (0.19 S/cm) is higher than that of Nafion^®^ 117 (0.113 S/cm) and PVA-ZrP (0.068 S/cm) [5]. The Nafion^®^/PVA-ZrP blend membrane’s proton conductivity has increased due to the increased concentration of the sulfonic acid moiety, which produces a porous structure and helps to retain more water [30]. According to the results in Table 1, the proton conductivity is improved when ZrP, DMSO, and GA are added to the PVA matrix. A continuous space for proton transport through the membrane is provided by the controlled water uptake, which also produces characteristics of moderate swelling.

## 4. Conclusions

The results shows that the addition of ZrP to the crosslinked PVA-ZrP membrane resulted in a remarkable improvement in the modulus of elasticity of 17,000 kPa compared to the value of 4700 kPa of Nafion^®^ 117 at a 10 mm/s strain rate. This showed that the modified membrane became stronger, more flexible, and able to withstand higher forces before breaking due to the crosslinking chemical process and incorporation of ZrP nanoparticles, which enhances the strength and stability of PVA. The reinforced PVA nanocomposite showed a higher thermal stability compared to the Nafion^®^ 117 membrane. This means that the composite membrane can withstand higher temperatures without significant degradation. This improvement is due to the presence of ZrP nanoparticles, which can enhance the thermal resistance of the material. It was found that the crosslinked PVA increased the water uptake capacity of the composite membranes. Furthermore, the conductivity of the composite membrane also improved with increases in the GA content. The addition of GA contributed to better ion conduction paths, resulting in an enhanced ionic conductivity. This increase in conductivity is particularly important for applications involving proton exchange membranes, such as in fuel cells, where ion conduction is crucial for efficient performance.

The composite membranes, containing both Nafion^®^ and PVA with crosslinking using GA, exhibited the highest water uptake capacities of 59% and 53%, which are significantly higher than the water uptake capacity of the commercial Nafion^®^ 117 membrane (34%). This finding indicates that the composite membrane has improved water retention properties compared to the commercial membrane. The results also show the increase in ion exchange capacity for the moderated membrane, which is an essential characteristic for fuel cell applications. Moreover, Nafion^®^/PVA-ZrP and PVA-ZrP nanocomposite membranes showed promise for use in fuel cells, with their high proton conductivity, good mechanical stability, and improved thermal stability.

## Figures and Tables

**Table 1:** The methanol permeability of Nafion^®^ 117 membrane and Nafion^®^/PVA-ZrP and PVA-ZrP nanocomposite membranes at 5M and 2M concentrations.

Methanol solutions	2 M	2 M	2 M	5 M	5 M	5 M
Temperature	30 °C	60 °C	80 °C	30 °C	60 °C	80 °C
Nafion 117	-	-	-	-	8.84 × 10^−7^	1.98 × 10^−6^
PVA-ZrP	-	-	-	-	-	1.01 × 10^−6^
Nafion^®^/PVA-ZrP	-	-	-	-	-	1.25 × 10^−6^

**Table 2:** The IEC and proton conductivity of Nafion^®^ 117 membrane and Nafion^®^/PVA-ZrP and PVA-ZrP nanocomposite membranes.

Membranes	Nafion^®^ 117	Nafion^®^/PVA-ZrP	PVA-ZrP
IEC (meq/g)	0.93	1.2	1.1
Proton conductivity (S/cm)	0.113	0.19	0.068
Water uptake % (30 °C)	30	38	40
Water uptake % (60 °C)	32	49	55
Water uptake % (80 °C)	34	53	59
Swelling ratio (30 °C)	9	23	25
Swelling ratio (60 °C)	26	28	30
Swelling ratio (80 °C)	29	34	37

## Data Availability

All data is contained within the article.
